# Transcriptome-based protein-protein interaction analysis reveals immune gene network elucidating white body immunity mechanisms in response to LPS stimulation in *Amphioctopus fangsiao*

**DOI:** 10.1016/j.cirep.2024.200151

**Published:** 2024-06-12

**Authors:** Zhengcai Lu, Yancheng Zhao, Tingjin Lv, Xipan Chen, Cuiju Cui, Xiumei Liu, Zan Li, Liyong Wang, Xiaohui Xu, Jianmin Yang

**Affiliations:** a School of Agriculture, Ludong University, Yantai 264025, PR China; b College of Life Sciences, Yantai University, Yantai 264005, PR China; c Yantai Institute of Marine Economy, Yantai 264003, PR China; d Yantai Marine Economic Research Institute, Yantai 264003, PR China

**Keywords:** *Amphioctopus fangsiao*, LPS, Innate immunity, Protein-protein interaction networks, White body, RNA-seq

## Abstract

One of the major challenges in the aquaculture of marine organisms is the susceptibility to pathogens. Immune cells defend bacterial invasion by recognizing pathogen-associated molecular patterns (PAMPs) such as Lipopolysaccharides (LPS) in Gram-negative bacteria. The white body are key organs for immune response and hematopoiesis, and play an important role in understanding the antimicrobial biological response in gold-ringed octopus (*Amphioctopus fangsiao*). In the present research, transcriptome sequencing and bioinformatics analysis were conducted on *A. fangsiao* white body tissue after 6 h and 24 h of LPS stimulation. The results showed 2,029 and 73 differentially expressed genes (DEGs) at the 6- and 24-hour time points, respectively. Gene Ontology (GO) and Kyoto Encyclopedia of Genes and Genomes (KEGG) enrichment analyses were beneficial in determining immune-related terms and signaling pathways. Subsequently, a protein-protein interaction (PPI) network was constructed and 20 hub genes, such as *AKT3, MAPK14*, and *PIK3CA*, were identified. These genes are involved in a variety of signaling pathways, including the Hippo signaling pathway, chemokine signaling pathway, and leukocyte trans endothelial migration. Using qRT-PCR, the accuracy of the 20 hub genes was verified. The present study provides a substantial theoretical foundation for the research on *A. fangsiao* white body immunity, enhancing the current understanding of cephalopods' innate immunity.

## Highlights

• This study integrates protein-protein interaction network into transcriptome analysis for the first time in cephalopods.
• The result lays a function for further exploring invertebrate LPS stress at the molecular level.
• It provides valuable gene resources for further study of the white body immune responses in pathogen-infected invertebrate.

## 1 Introduction

Invertebrates possess a sophisticated immune system, which has garnered significant scholarly attention [1]. As one of the immune organs in cephalopods, white body were discovered as early as 1984 in Northern shortfin squid (*Illex illecebrosus*) and were considered as hematopoietic organs in cephalopods in subsequent studies, which may be involved in important functions such as immune regulation, hematopoietic activation, and pathogen infection [2]. For example, the white body of cephalopods contain blood cells at different stages of development [3]. In addition, white body obtain arterial blood from several branches of the ophthalmic artery and form a complex network of capillaries within the organ [4]. This network not only contributes to the maturation of blood cells, but also facilitates the entry of mature blood cells into the circulatory system through the capillaries, thereby participating in immune responses and other immune activities. Hemocytes in the white body are produced by leukocytes, which play a key role in a variety of cellular immune processes including phagocytosis, lysis, and attack, thus becoming an important component of the innate immune system [3,5].

The cephalopod Gold-ringed octopus (*Amphioctopus fangsiao*), popular among consumers for its flavorful meat and high arginine content, is a traditional dietary in coastal areas of China [6,7]. There has been a recent surge of interest in the artificial cultivation of gold-ringed octopus, *A. fangsiao*, but relevant research endeavors have been considerably hindered by disease outbreaks brought on by pathogenic infestations [8,9]. Seawater hosts a majority of gram-negative bacteria [10], several species of which have been identified to be harmful to various marine organisms such as Pacific oyster (*Crassostrea gigas*) [11], Horsehair crab (*Portunus trituberculatus*) [12], Japanese flounder (*Paralichthys olivaceu*) *s* [13], Pacific white shrimp (*Penaeus vannamei*) [14], and Green-lipped mussel (*Perna canaliculus*) [15]. Lipopolysaccharide (LPS) is a major component of the cell wall of Gram-negative bacteria and consists of O-antigens, core oligosaccharides, and covalently linked lipid A, which is the main active center of LPS [16]. In the host's immune system, LPS is recognized by immune cells as pathogen-associated molecular patterns (PAMPs) and initiates the immune response process upon invasion by Gram-negative bacteria [17]. This recognition process triggers immune signaling pathways, which in turn stimulate host defense mechanisms.

RNA-seq presents a potent approach towards deciphering gene transcription profiles inherent to specific biological processes [18,19]. Recently, this technique has proven valuable in investigating the molecular mechanisms crucial for immunity [20], culminating in the discovery of a myriad of immunity-related genes across diverse organisms such as Jamaican fruit bat (*Artibeus jamaicensis*) [21], Yesso scallop (*Patinopecten yessoensis*) [22], and Bearded fireworm (*Hermodice carunculata*) [23]. Previous studies on Gram-negative bacterial infections have typically focused on individual genes [[24], [25], [26]]. While these single-gene studies enrich scientific knowledge, their scope of research remains inherently restrictive. Notably, genes do not act independently; instead, they frequently participate in numerous regulatory pathways, influencing cellular processes through the formation of expansive functional networks [27]. Creating an immune interaction network to identify crucial network nodes provides a strategic method for pinpointing hub genes that are instrumental in immune responses.

In the present study, the transcriptional profiles of *A. fangsiao*'s white body tissue were examined following LPS injection stimulation, with assessments conducted at time points of 6 and 24 hours. Transcriptome sequencing was performed using the Illumina HiSeq 6000 platform, and the transcriptome data were subsequently screened for differentially expressed genes using the DESeq2 method based on negative binomial distribution. Through GO and KEGG functional enrichment analyses, we dug deeper into the important genes involved in the *A. fangsiao* immunization process. In addition, we constructed protein-protein interaction (PPI) networks to identify hub DEGs associated with LPS infection. Finally, the screened 20 hub genes were verified by qRT-PCR to ensure their consistency with the RNA-Seq results. These findings provide important clues to our in-depth understanding of the immune response of *A. fangsiao*, and also provide a strong reference for future related studies, thus promoting the study and understanding of the function of the cephalopod immune system.

## 2 Material and Method

### 2.1 Sample preparation

We collected *A. fangsiao* specimens from the Yellow Sea area of Yantai City, Shandong Province, China. To ensure the stability of the samples during the experiment, we placed the specimens in five temporary tanks, each measuring 70.4 cm in length, 52.5 cm in width, and 87.4 cm in height. These tanks are filled with oxygen-rich seawater and are maintained in a temperature range of 18 to 20 °C. The water quality indicators for farmed seawater include: dissolved oxygen = 5.4 mg/L, pH = 8.2, and salinity = 30 ± 0.5 ppt. The qualitative indicators remained stable throughout the experiment; this was also true for the seven days prior to the start of the experiment. To mitigate potential stress, we put food and ceramic jars in the tank. Healthy, vigorous specimens of *A. fangsiao* with an average age of about five months and an average weight of 34.7 g were purposely selected for subsequent experiments. The experimental samples were divided into three groups, a blank group without any treatment (AB), a control group injected with 100 μLPBS (AP), and an experimental group injected with the same volume of LPS (AL). The injections were administered at the first left wrist, where it touched the wrist. Group AB used sterilized dissection tools to dissect carcass parts of Fanshawe pigs Group AB used sterilized dissection tools to dissect carcass parts of *A. fangsiao*, removed white body tissues, and immediately froze them in liquid nitrogen and stored them at -80 °C for subsequent analysis. The same operations of two other groups happened at 6 h and 24 h post-injection, respectively.

### 2.2 The extraction of RNA

In each of the above three treatment groups, nine gold-ringed octopuses were randomly selected for RNA extraction: 0 h sampling (C0h) for group AB, 6 h sampling (P6h) for group AP, 24 h sampling (P24h) for group AP, 6 h sampling (L6h) for group AL, and 24 h sampling (L24h) for group AL.From the nine *A. fangsiao* specimens, equal molar masses of three random samples were combined to create a single replicate [28]. This process was repeated three times, resulting in the formation of three repetitions, which served as the template for the construction of the transcriptome library. The procedure was then duplicated to procure two extra replicates.

Using the Trizol method, total RNA extraction was conducted [29]. The prepared tissues were treated with Trizol reagent. After standing, they were centrifuged and mixed with chloroform. The aqueous layer was then separated and isopropanol was added to induce the formation of a precipitate. The quality and quantity of total RNA were weighed up by 1.5 % agarose gel electrophoresis and a microspectrophotometer. Quality control of RNA was performed by means of the Agilent 2100 bioanalyzer [30].The surplus RNA was carefully stored for subsequent quantitative real-time PCR validation.

### 2.3 Library construction and sequencing

The acquired total RNA was enriched for mRNA with polyA tails by Oligo (dt) magnetic beads. Subsequently, these isolated mRNAs underwent random fragmentation using divalent cations. This step laid the foundation for constructing a library, employing the standard library construction method from NEB. The first strand of cDNA was synthesized in the M-MuLV reverse transcriptase system using fragmented mRNA as a template and random oligonucleotides as primers, followed by degradation of the RNA strand with RNaseH and synthesis of the second strand of cDNA with dNTPs in the DNA polymerase I system. Following the purification of double-stranded cDNA, it underwent end-repair, A-tailing, and was connected to the sequencing junction. The cDNA fragments ranging from 250 to 300 bp were selected using AMPure XP beads. PCR amplification was carried out, and the resulting PCR products were purified once more using AMPure XP beads to ultimately obtain the library. The library construction kit was NEBNext® Ultra™ RNA Library Prep Kit for Illumina®. The Agilent 2100 BioAnalyzer served to ascertain the insert size, which guaranteed the library's quality. In the final stage, the library was subjected to sequencing, which was executed on the Illumina NovaSeq 6000 platform.

### 2.4 Splicing and functional annotation of transcripts

Quality control analysis yielded clean reads, which were subsequently assembled via Trinity to produce reference sequences. The quality of the sequences was evaluated using BUSCO software [31]. Upon obtaining spliced transcripts, gene function annotations were performed against six different databases: Swissprot, Nr, GO, KEGG, KOG/COG, and Nt.

### 2.5 Mapping and differential expression analysis

The Reference Sequence-based Estimation of Expression Model (RSEM) was utilized to align the purified reads of each specimen to the reference sequences. The number of reads that were mapped to each gene was designated as the read count. This read count was later converted to FPKM to approximate the levels of gene expression. DEGs were identified by employing DESeq2 software [32]. The criteria used for identification included a *q*-value of less than or equal to 0.05 and an absolute value of log2 fold change greater than or equal to 1.

### 2.6 Function enrichment analysis

Functional enrichment analysis was performed on the DEGs. To determine the GO terms and the distribution of DEGs, GO analyses were deployed on the union set distinguished at two distinct time points. In addition, Gene Set Enrichment Analysis was employed to identify immune-related pathways and genes through the KEGG pathway analysis, thereby elucidating the functions of DEGs. Enrichment analyses of GO and KEGG were executed using the DAVID database (https://david.ncifcrf.gov/) 2021 [33].

### 2.7 Construction of protein interaction network

Creating a PPI network can provide valuable insights into the relationships among immune pathways, facilitating the identification of key genes. In the present study, the STRING database (https://cn.string-db.org) was employed to construct a robust PPI network.

### 2.8 Validation of RNA-Seq

In the present study, 20 hub genes were highlighted for validation via qRT-PCR. The primary reference factor for identifying hub genes was the count of DEGs involved in interactions within the PPI network. This count was combined with the number of DEGs participating in the enriched KEGG signaling pathway. Utilizing Primer Premier 5.0 software, gene primer sequences were formulated based on the spliced transcriptome. Table 1 provides the sequence details for the 20 primers. The *β-actin* gene served as the reference due to its evident stability within the experiment. The fluorescence quantification methods implemented were adapted from Bao et al.'s research [34].

**Table 1:** List of primers used for quantitative RT-PCR validation.

Gene name	Official Gene Name	Forward primer (5′-3′)	Reverse primer (5′-3′)	Amplicon length (bp)
*AKT3*	AKT serine/threonine kinase 3	GATAAACCTCGGCCCAATAC	GCATCCATTCTTCCCTTTCT	106
*CASP3*	caspase 3	GTGCCCAGGTTTAATAGGTAAG	GATGCTTGACTCTGACATCAC	113
*CASP7*	caspase 7	GAAAGTGGTAAGGGTCTGTATC	AGTCACCTCCCTTGTCTTTA	106
*CASP8*	caspase 8	CGGGCATTACGGAACTTT	GTGCTTTCCTCAGCTTCTT	109
*CDKN1B*	cyclin dependent kinase inhibitor 1B	TCGAGTTAGACACAGTAGGG	CGGTAATAACGACGACAACA	119
*HDAC2*	histone deacetylase 2	ACGATCTCCCGACAAAGA	GGTATCGATGATGAGTCGTATG	120
*ITGA6*	integrin subunit alpha 6	TCGGCTGTGAGCATAGT	CAGCACCATCACCTTCATAG	111
*LIMK2*	LIM domain kinase 2	AAAGCGGAGAAGCAAGTC	GGTCACTTGAGCGAAACA	122
*MAPK14*	mitogen-activated protein kinase 14	CTGGAGCAAGTCTCTGATAAC	GCGAATGAGATGGCTACTG	103
*NOTCH2*	notch receptor 2	GTCTCGGCACAGGTTATTC	ATCCTGGACAGTCGTAAGT	129
*PIK3CA*	phosphatidylinositol-4,5-bisphosphate 3-kinase catalytic subunit alpha	CTCAGCCACTTCACTTCTATC	GTCAGCCGAGATCCTTTATC	144
*PKM*	pyruvate kinase M1/2	GCGTTATCCAGCGTGATTT	GCTGTGGCTCTAGACACTAA	116
*PLCG1*	phospholipase C gamma 1	GAGTGGCAGAGAGAGATAGA	CGGGCTAGAAGAAGAAGAAG	117
*PPP1CA*	protein phosphatase 1 catalytic subunit alpha	CCAGCCCATTGTTTCCTT	GGTGGTTTGAGTCCTGATTT	129
*PRKCI*	protein kinase C iota	ACCAAACAACCCTTCCTTAC	CTCTGGCCCTTAACTTTCTAC	131
*PTPN11*	protein tyrosine phosphatase non-receptor type 11	CGAGACGAAAGAACTGGATG	CCTTCTCGGACCTTCTATCT	136
*RAC1*	Rac family small GTPase 1	CCTGAGAGAAGACAAGGAAAC	CGTCAAAGCAGAGCATTCTA	130
*SMAD4*	SMAD family member 4	TGCTGTTCACCAACATAGG	CCATCTGGGCCACAATTTA	115
*VAV1*	vav guanine nucleotide exchange factor 1	CAACAAGTTCTGGGAGTGAG	ACACGAGCAGGAGAGTTAT	133
*YAP1*	Yes1 associated transcriptional regulator	CCCATCTTGTGTGTCCATTT	CTACCAATCAGGCAGACCTA	134

## 3 Results

### 3.1 Sequencing and mapping

The sequencing process was administered for five distinct time points, each comprising three replicates namely, C0h_1, C0h_2, C0h_3, P6h_1, P6h_2, P6h_3, P24h_1, P24h_2, P24h_3, L6h_1, L6h_2, L6h_3, L24h_1, L24h_2, and L24h_3. The pertaining statistical data is presented in Table 2. An examination of the results indicates an average of 21,914,359 raw reads compared to 21,253,768 clean reads. The mean percentages for Q20 and Q30 were recorded as 96.52 % and 91.37 % respectively. The GC content exhibited an average of 38.57 %, whereas the average mapping rate was duly noted to be over 70 %.

**Table 2:** List of sequencing information.

Sample	Raw reads	Clean reads	Q20( %)	Q30( %)	GC pct	Total mapped
C0h_1	19,939,253	19,287,831	96.67	91.83	38.68	72.98 %
C0h_2	20,418,836	19,863,968	97.06	92.51	39.33	77.38 %
C0h_3	23,818,581	22,964,042	95.79	89.48	37.62	71.47 %
L6h_1	22,177,842	21,439,506	96.94	92.34	39.97	75.43 %
L6h_2	22,621,027	22,034,160	97.22	92.71	38.45	75.39 %
L6h_3	21,314,204	20,641,328	96.88	92.13	39.81	76.80 %
P6h_1	22,264,350	21,627,478	96.59	91.72	37.83	71.20 %
P6h_2	20,337,942	19,774,901	96.20	90.74	37.28	69.91 %
P6h_3	23,364,882	22,734,613	96.00	89.85	37.49	71.06 %
L24h_1	20,552,220	19,800,202	96.76	91.91	39.51	74.87 %
L24h_2	23,732,660	23,015,373	95.41	88.70	37.73	70.48 %
L24h_3	20,651,203	20,056,680	96.47	91.50	37.46	70.03 %
P24h_1	22,661,079	22,055,170	96.14	90.75	38.00	70.67 %
P24h_2	23,213,684	22,426,500	97.00	92.36	40.58	77.10 %
P24h_3	21,647,624	21,084,780	96.77	92.07	38.86	73.51 %
Average	21,914,359	21,253,768	96.53	91.37	38.57	73.22 %

### 3.2 Screening of differential genes and expression analysis

**Fig. 1:**
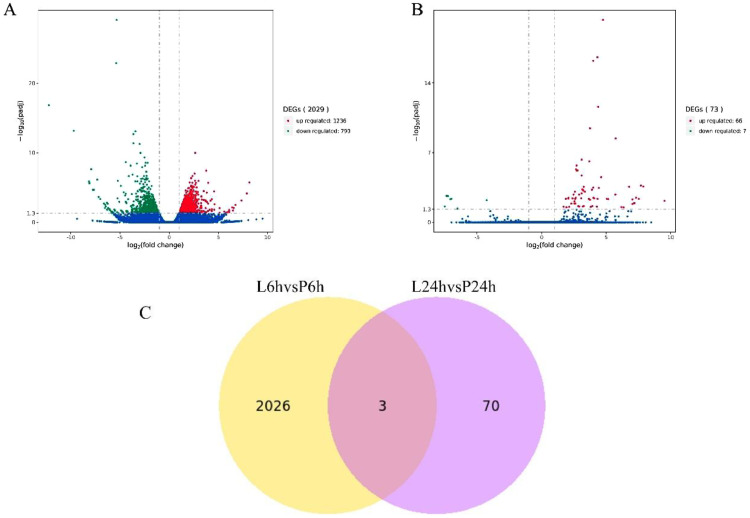
Distribution and number of DEGs. (A) The volcano plot of differential gene expression measured at 6 hours. The red dots denote upregulated DEGs, the green dots denote downregulated DEGs, and the blue dots denote genes having no differential expression. (B) The volcano plot of differential gene expression captured at 24 hours. (C) Venn diagrams for DEGs. The yellow areas indicate the number of DEGs significantly differentially expressed at 6 h, the purple areas indicate the number of DEGs significantly differentially expressed at 24 h, and DEGs significantly differentially expressed at all time points are shown in dark red.

**Fig. 2:**
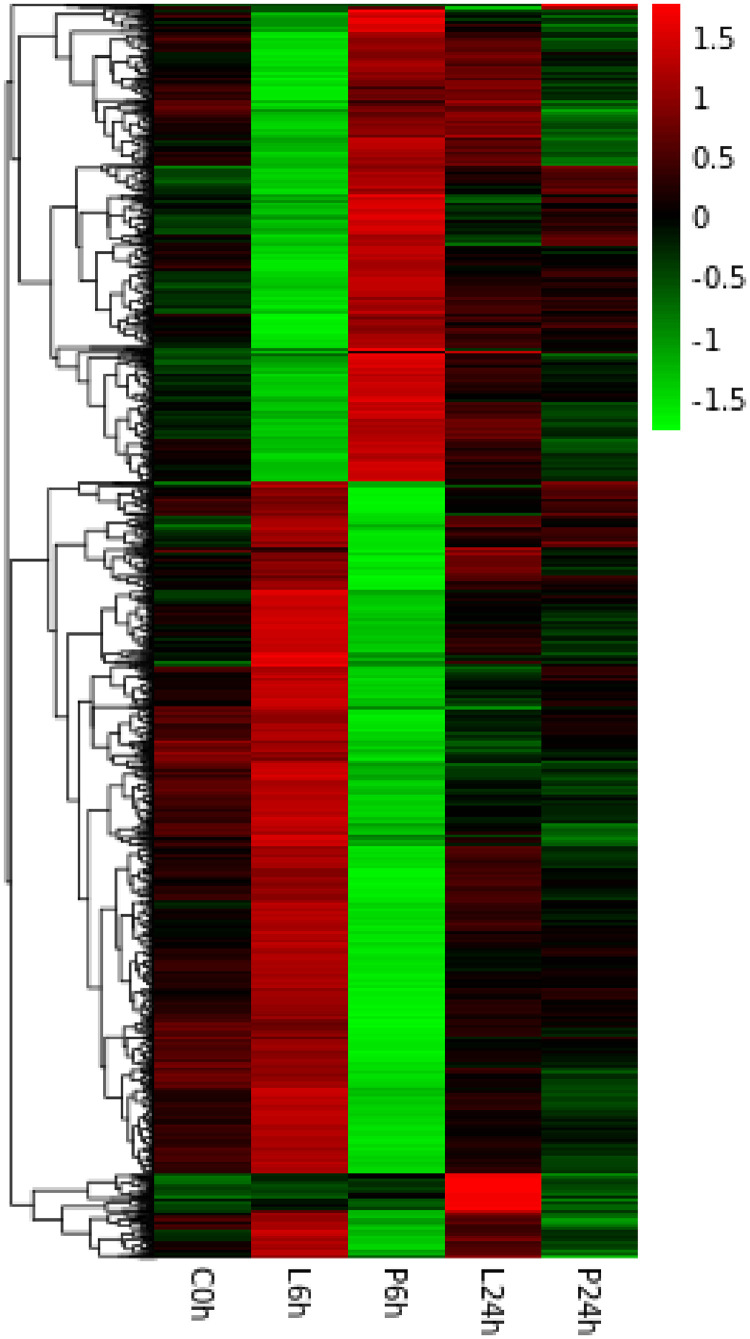
Hierarchical clustering heatmap of the DEGs. The x-axis signifies the distinct time points, each denoted by a unique horizontal line representing an individual gene. A color gradient extending from red to green indicates a transition from high to low expression levels of DEGs, respectively.

Following LPS stimulation, a notable number of DEGs were detected, comprising 1236 upregulated and 793 downregulated genes within a 6-hour timeframe. After 24 hours, the count decreased to 66 upregulated and 7 downregulated DEGs. The total number of genes displaying significant differential expression was 2099 (*q*-value ≤ 0.05, |Log2FoldChange| ≥ 1) (Table S1–4). The distribution of such genes is clearly detailed in the volcano plots shown in Fig. 1A and 1B. Post-injection, an intersection of 3 genes was observed between the entire set of 2102 genes. The Venn Diagram shown in Fig. 1C demonstrates the overlap between the two-time frames. The focus of subsequent analysis was on the unified set of DEGs, taking into account both time points due to their potential integral role in immunological functions. The 2099 DEGs were further utilized for the hierarchical clustering heatmap analysis, and the differential gene expression profiles are depicted in Fig. 2.

### 3.3 GO and KEGG functional enrichment analyses

**Fig. 3:**
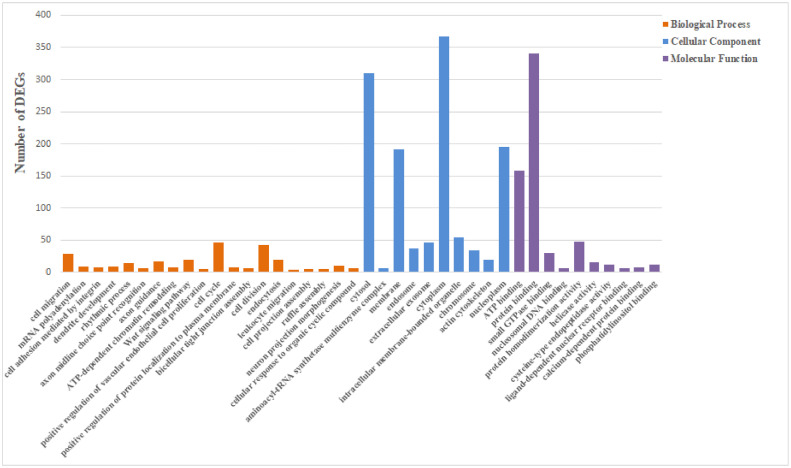
The GO enrichment analysis of DEGs. The X-axis illustrates the GO terms, and the Y-axis signifies the quantity of DEGs. Each category is represented by different colored bars: the biological process is shown in orange, the cellular component in blue, and the molecular function appears in magenta.

**Fig. 4:**
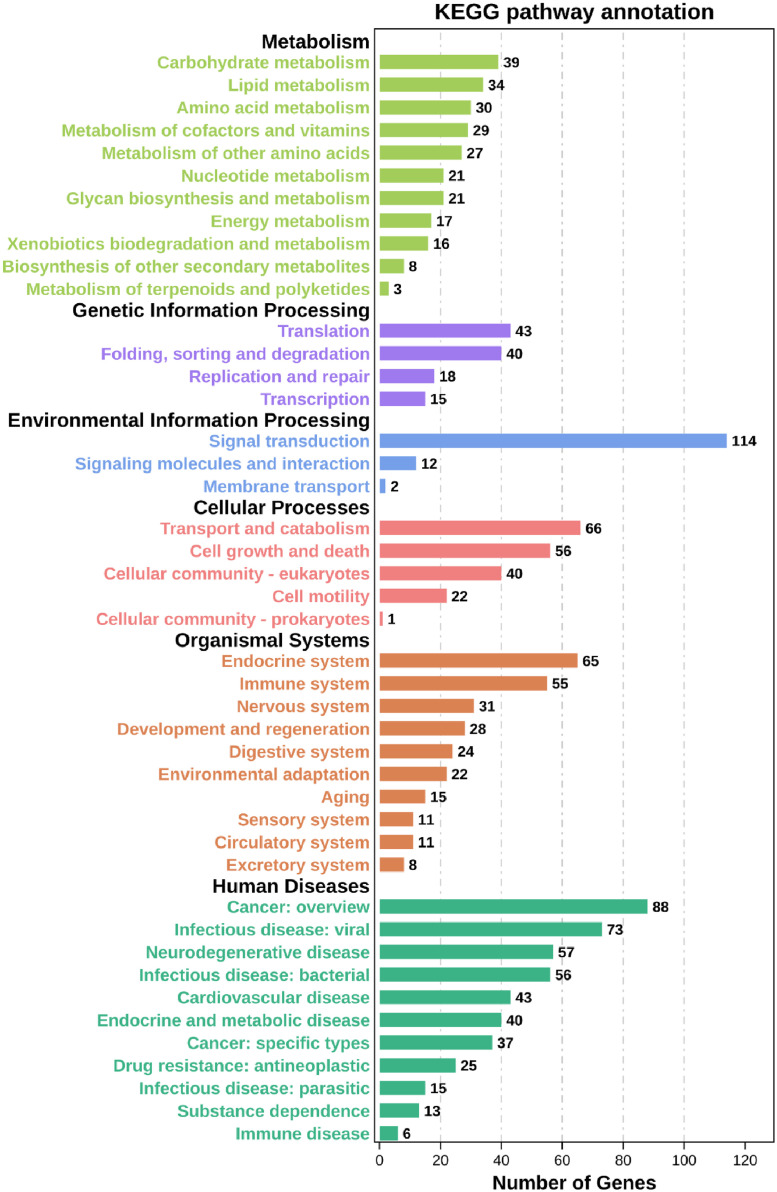
Distribution of level-2 KEGG classes. The horizontal axis denotes the count of DEGs, while the vertical axis signifies specific level-2 KEGG classes. Level-2 is the second level of categorization of the KEGG database, which enables a more systematic presentation of the biological processes in which DEGs are involved.

The task of performing GO functional and KEGG pathway enrichment analyses was carried out on a pool of 2099 DEGs. This GO annotation analysis yielded 657 level-3 subcategories within the Biological Process category, 173 under the Cellular Component category, and 217 in the Molecular Function category. Level-3 is the third level of categorization of the GO database and enables a more detailed presentation of the specific functions undertaken by DEGs. Fig. 3 depicts the leading 20 Biological Process categories coupled with the top 10 subcategories from each aforementioned category. Illustrated in Fig. 4 is the level-2 KEGG diagram. As clearly showcased in Fig. 4, a significant portion of the DEGs were associated with the immune system. Subsequently, an analysis was conducted to identify 23 immune-related signaling pathways for a more comprehensive examination (Table 3).

**Table 3:** A summary of 23 immune pathways.

Signaling pathway name	Number of DEGs
Pathways in cancer	17
Lysosome	8
Transcriptional misregulation in cancer	8
Endocytosis	7
PI3K-Akt signaling pathway	6
Central carbon metabolism in cancer	5
Chemical carcinogenesis - reactive oxygen species	5
MicroRNAs in cancer	5
Proteoglycans in cancer	5
Chemokine signaling pathway	4
Fc epsilon RI signaling pathway	4
Fc gamma R-mediated phagocytosis	4
Inflammatory mediator regulation of TRP channels	4
Leukocyte transendothelial migration	4
T cell receptor signaling pathway	4
Toll-like receptor signaling pathway	4
Choline metabolism in cancer	3
Hippo signaling pathway	3
Natural killer cell mediated cytotoxicity	3
PD-L1 expression and PD-1 checkpoint pathway in cancer	3
Th1 and Th2 cell differentiation	3
VEGF signaling pathway	3

### 3.4 PPI network analysis

**Fig. 5:**
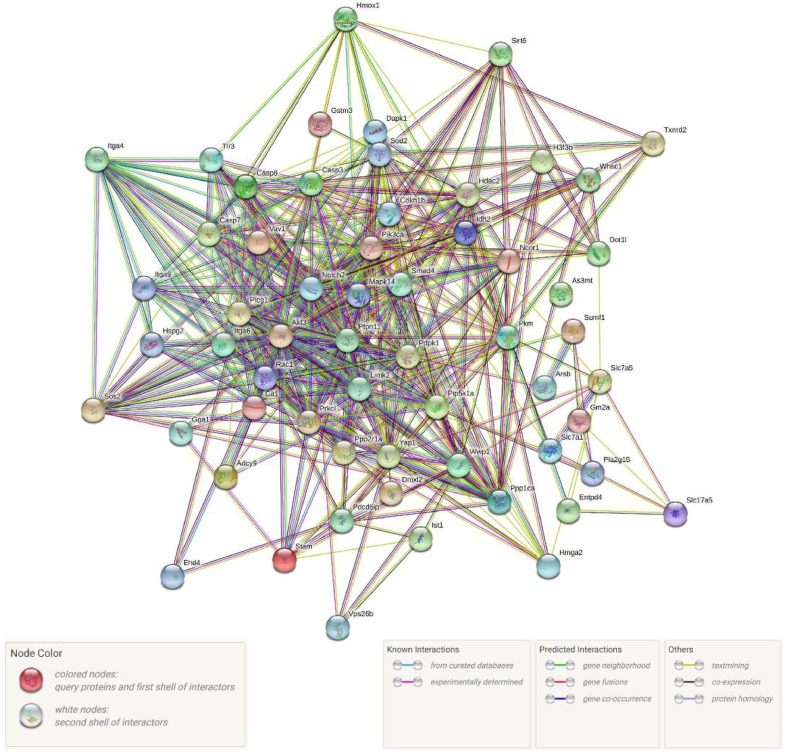
The immune-related protein interaction network. Each node represents a protein, and the lines between the nodes represent that they have an interaction relationship. The number of lines between the nodes represents the strength of the interaction.

The elaborately constructed protein association network is demonstrated in Fig. 5 which elucidates light on the intricate relationships between immune genes. Sixty DEGs involved in 23 signaling pathways closely related to the immune response were used to construct the PPI. Table 4 provides an in-depth description of the network's elements.

**Table 4:** A summary of the PPI network.

Network Stats	
Number of nodes	59
Number of edges	487
Expected number of edges	357
PPI enrichment *P*-value	4.87e-11
Average node degree	16.5

### 3.5 Verification of the RNA-seq accuracy using qRT-PCR

**Fig. 6:**
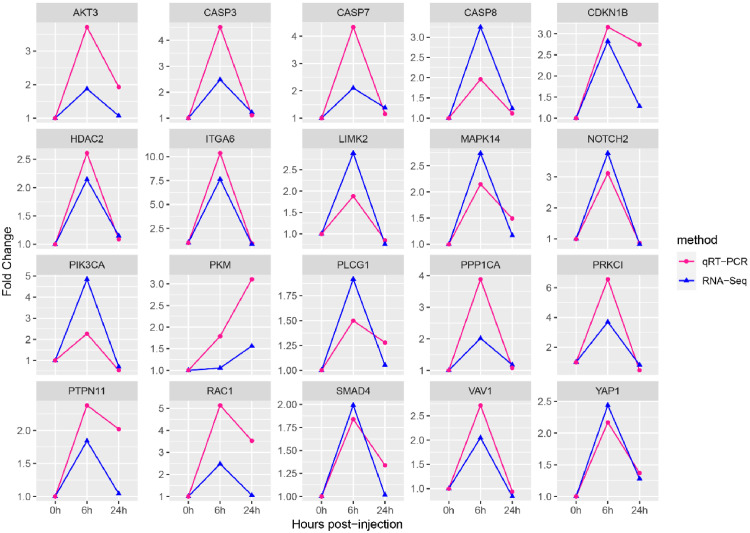
Comparison of the 20 hub DEGs between qRT-PCR and RNA-seq expression levels. The horizontal axis signifies the time point post Lipopolysaccharide (LPS) stimulation, and the vertical axis illustrates the fold change. The same trend of change at different time points represents that the RNA-Seq results are accurate.

The validation of the 20 hub genes, as outlined in Table 5, was conducted through qRT-PCR. A comparative analysis was conducted between RNA-seq and qRT-PCR, utilizing fold change values. Fig. 6 demonstrates a consistent pattern in the observed expression trends between qRT-PCR and RNA-seq.

**Table 5:** Statistics of 20 hub genes.

Gene name abbreviation	Gene name official full name	Number of protein interaction	Number of KEGG pathways
*AKT3*	AKT serine/threonine kinase 3	38	3
*MAPK14*	mitogen-activated protein kinase 14	36	9
*PIK3CA*	phosphatidylinositol-4,5-bisphosphate 3-kinase catalytic subunit alpha	36	13
*CASP3*	caspase 3	34	2
*RAC1*	Rac family small GTPase 1	33	1
*CDKN1B*	cyclin dependent kinase inhibitor 1B	32	3
*LIMK2*	LIM domain kinase 2	30	1
*HDAC2*	histone deacetylase 2	28	3
*PKM*	pyruvate kinase M1/2	28	1
*PRKCI*	protein kinase C iota	28	1
*SMAD4*	SMAD family member 4	28	1
*CASP8*	caspase 8	27	2
*PTPN11*	protein tyrosine phosphatase non-receptor type 11	27	1
*YAP1*	Yes1 associated transcriptional regulator	27	1
*NOTCH2*	notch receptor 2	26	2
*CASP7*	caspase 7	24	1
*ITGA6*	integrin subunit alpha 6	23	2
*PLCG1*	phospholipase C gamma 1	23	12
*VAV1*	vav guanine nucleotide exchange factor 1	22	1
*PPP1CA*	protein phosphatase 1 catalytic subunit alpha	21	1

## 4 Discussion

### 4.1 The clustering and enrichment analyses of DEGs

We first screened a set of DEGs based on transcriptomic data at the two investigated time points and performed a heatmap analysis using the concatenated set of DEGs (2099) at these two time points. Heat map analysis showed significant clustering and expression pattern differences between the AL group and the AP group, which further validated the significant effect of LPS stimulation on gene expression patterns. In addition, although there were small differences between theAP and AB groups, they may be related to the effect of the injection stimulus on the immune response. GO and KEGG functional enrichment analyses further confirmed that immune responses were significantly activated in *A. fangsiao* after LPS stimulation. For example, a large number of GO terms related to the immune response were enriched, including the Wnt signaling pathway, endocytosis, positive regulation of vascular endothelial cell proliferation and leukocyte migration, were enriched in biological processes [[35], [36], [37]]. Similarly, various immune-associated KEGG signaling pathways, including chemokine signaling pathway, Hippo signaling pathway, and leukocyte transendothelial migration were identified in the KEGG level-3 subclass [[38], [39], [40]]. However, a gradual decrease in the number of significantly DEGs was observed over time, possibly suggesting that the intensity of LPS stimulation was too high, leading to a failure of immune defenses. In order to further expound the mechanisms of leucosomal immunity to LPS stimulation in *A. fangsiao*, an in-depth exploration of the functions of these genes, as well as the effects of LPS stimulation on immune defense functions, is necessary.

### 4.2 Identification of the hub genes

Proteins, as primary facilitators of diverse biological processes, regulate life processes such as metabolism, signal transmission, cell cycle, and gene expression [41,42]. In the present study, a protein interaction network was constructed based on 60 DEGs derived from immune-related pathways. We found that the proteins in this network exhibit a higher degree of interconnectivity with each other compared to a randomly selected set of similar size, which further confirms the biological importance and relevance of the proteins we studied. We hypothesize that the hub proteins in these networks may have a crucial role in immune function because of their high number of interactions in the network. Therefore, we defined proteins with a high number of interactions as hub proteins and designated their corresponding genes as hub genes.

### 4.3 Discussion of hub genes and pathways

Through PPI analysis, we successfully predicted and screened 20 hub genes with multiple interactions that play important roles in immune regulation. These genes were categorized into eight specific functional groups encompassing multiple immune-related pathways, including the Caspase family, the Hippo signaling pathway, the Fc epsilon RI signaling pathway, the Chemokine signaling pathway, Inflammatory mediator regulation of TRP channels, Natural killer cell-mediated cytotoxicity, Leukocyte transendothelial migration, and the VEGF signaling pathway. These findings further reveal the complexity and diversity of white body immune mechanisms in *A. fangsiao* in response to LPS stimulation.

#### 4.3.1 Caspase family

The essential role of apoptosis, also referred to as programmed cell death, in development and maintaining tissue homeostasis is of paramount importance [43,44]. Serving a central function in apoptosis and inflammatory response management is the caspase family, which facilitates the elimination of infected cells, the maintenance of internal environmental stability, and immunity enhancement [[45], [46], [47], [48]]. As is well-documented, caspase family genes, such as *CASP1* [49], *CASP4* [50], *CASP5*, and *CASP11* [51], are influential in immune regulation. In the present research, *CASP3, CASP7*, and *CASP8* as key genes involved in significant immune processes in the *A. fangsiao* species. *CASP3*, in particular, plays a pivotal role in the execution phase of programmed cell death and has recently been recognized as a mediator of inflammation [52]. *CASP7* is linked closely with immune cells such as neutrophils and dendritic cells [53], playing a significant role in apoptosis [54]. Similarly, *CASP8* regulates pyroptosis, necroptosis, and apoptosis as a molecular checkpoint, mitigating tissue damage by managing inflammatory response [55]. An analysis of their expression levels revealed a significant upregulation at the six-hour time point, followed by a decline at the 24-hour mark. We speculate that the dynamic expression of *CASP3, CASP7*, and *CASP8* genes after LPS stimulation may reflect their different stages of roles in the *A. fangsiao* immune response. The upregulation at 6 h may be a rapid response after inflammatory infection to clear infected cells and maintain immune homeostasis by regulating apoptosis and inflammatory responses. In contrast, the marked decline after 24 h may reflect a gradual waning of the inflammatory stimulus and a regulatory and recovery phase of the immune response. This is an important manifestation of the stress immune response of *A. fangsiao* against bacterial infection.

#### 4.3.2 Hippo signaling pathway

The Hippo signaling pathway has been demonstrated to be crucial in maintaining homoeostasis of the immune system and regulating the size of immune organs [56]. Such pathway regulates apoptosis, cellular proliferation, and stem cell self-renewal, impacting tissue homoeostasis, organ size, and tissue remodeling [57]. Further, recent research has demonstrated the Hippo pathway's role in regulating innate immunity [58]. During the present investigation, findings were made that several critical genes - *PRKCI, YAP1*, and *PPP1CA* were enriched in this pathway. *PRKCI* is involved in activating NF-kappa-B and provides a broad protective effect against apoptotic stimuli [59,60]. Such mechanism aids in damage protection by mitigating excessive immune responses. *YAP1* activates the transcription of pro-inflammatory cytokines, which recruit and expand immune cells. Moreover, studies have suggested that *PRKCI* promotes immune suppression via *YAP1* [61]. *PRKCI* is one of three catalytic sub-units for protein phosphatase 1, which negatively regulates RIG-I-like receptors and Toll-like receptors to balance the immune system [62]. In the present study, these three critical genes exhibited significant up-regulation at six hours, with a decrease observed at twenty-four hours. We hypothesized that they maintain immune system homeostasis by altering transcript expression levels in response to LPS stimulation. For example, the immune function of *A. fangsiao* white body after bacterial stimulation is enhanced by mechanisms such as anti-apoptosis, anti-injury, and receptor control.

#### 4.3.3 Chemokine signaling pathway and Leukocyte transendothelial migration

Chemokines, a sub-group of small cytokines, aid the recruitment of various leukocyte subsets and direct leukocyte migration to definitive locations during inflammatory responses. In parallel, they are indispensable for facilitating interactions among immune cells [63,64]. The transendothelial migration of leukocytes, essential for triggering both innate and adaptive immune responses, considerably impacts immune surveillance across organisms [40,65]. In our study, we observed that LPS stimulation may be mediated by activation of chemokine signaling pathways and leukocyte transendothelial migration signaling pathways. This may lead to the release of cellular signals and factors that trigger the migration of immune cells such as leukocytes and macrophages toward the site of infection or injury. At the same time, the synergistic effect of these processes helps accelerate the localization and aggregation of immune cells at the site of infection or injury. This in turn accelerates the initiation of the inflammatory response and the clearance of the foci of infection.Furthermore, Phospholipase C Gamma 1 (*PLCG1*) was identified as a central gene enriched in these two signaling pathways. Acting as an information conduit, *PLCG1* controls intracellular signaling cascades, actin reorganization, and cellular migration [66]. A significant upregulation of this gene was observed at 6 hours, which subsequently declined at 24 hours. This fluctuating expression pattern significantly enhanced immune cell migration and communication, thereby strengthening resistance to bacterial invasion in the white body tissue of *A. fangsiao*. In summary, the Chemokine signaling pathway and Leukocyte transendothelial migration was critical to the immune system of *A. fangsiao* white body. However, the specific molecular mechanism and biological effects still need to be confirmed by further experimental studies.

#### 4.3.4 Top three hub genes

In the present research, protein interactions were examined to determine hub genes, and *AKT3, MAPK14*, and *PIK3CA* were ultimately identified as the most predominant based on strong protein-protein interactions. *AKT3*, from the serine/threonine kinase family, significantly impacts various biological regulation processes [67]. Notably, it modulates cell proliferation, and inhibiting *AKT3* leads to cell cycle cessation in the G1 phase [68]. Moreover, *AKT3* is associated with the viability of immune cell types such as monocytes and macrophages [69]. The *MAPK14* gene, a member of the MAP kinase family, serves as an innate immune system modulator, particularly activating macrophages. Similarly, *MAPK14* plays a vital role in immune response cascades triggered by external stimulation, such as physical stress and inflammatory cytokines, and directly activates transcription factors [[70], [71], [72], [73]]. *PIK3CA*, an essential component of the PI3K signaling pathway, recruits proteins that possess pH domains to the cell membrane, initiating the signaling cascade responsible for processes such as proliferation, cell growth, and survival [74,75]. The PI3K/AKT signaling pathway crucially regulates various cellular processes [76,77]. In the present study, *AKT3* and *PIK3CA* were identified as hub genes with significant 6 h upregulation, indicating their critical immune function via the PI3K/AKT cascade pathway. For example, it can regulate cell proliferation and growth, and participate in the PI3K/AKT signaling pathway to regulate immune cell activation and promote the initiation of immune response. At the same time, it regulates cell survival and apoptosis and helps maintain immune homeostasis and tissue homeostasis. *MAPK14* also exhibited significant upregulation at 6 hours, followed by a decrease at 24 hours. This underscores the importance of these top hub genes in *A. fangsiao*'s immune responses. Specifically, processes such as cell proliferation, pro-inflammatory responses, and immune cell activation play significant roles in *A. fangsiao*'s white bodies. This underscores the need for future in-depth investigations to elucidate their specific mechanisms.

#### 4.3.5 Other hub genes and pathways

The observed results encompass several pathways and genes that have not been previously mentioned. These include the Fc gamma R-mediated phagocytosis, the regulation of TRP channels by inflammatory mediators, the VEGF signaling pathway, cytotoxicity mediated by natural killer cells, and the involvement of *NOTCH2, RAC1*, and *SMAD4* genes. Fc gamma R-mediated phagocytosis are classical inflammatory or immune-related signaling pathways that can be activated by LPS stimulation [78]. TRP channels are ion channels on cell membranes that play an important role in immune regulation. Modulation of TRP channels by inflammatory mediators may affect intracellular calcium ion concentration and cellular activation, which are involved in the regulation and transmission of inflammatory responses [79]. VEGF is involved in the regulation of inflammation and immune responses by modulating angiogenesis and permeability, as well as promoting migration and activation of immune cells [80]. Natural killer cells are important components of the immune system that recognize and kill infected and tumor cells [81]. We hypothesize that after LPS stimulation, *A. fangsiao* leukosomes may act synergistically through Fc gamma R-mediated phagocytosis and cytotoxicity mediated by natural killer cells to jointly clear infected cells and pathogenic microbes. Regulation of TRP channels by inflammatory mediators may affect vascular endothelial cell function and vascular endothelial growth factor signaling pathway activity. This may influence the migration of immune cells and the development of tissue inflammation.Proteins encoded by the *NOTCH2* play a key role in cell signaling and are involved in the regulation of cell proliferation, differentiation and fate decisions [82]. *Rac1* belongs to the Rho GTPase family .It acts as an intracellular signal transducer involved in the regulation of various biological processes such as cytoskeletal organization, gene transcription and cell proliferation [83]. *SMAD4* is a key factor regulating TGF-β signaling and plays an important role in regulating biological processes such as cell proliferation, differentiation and apoptosis [84]. In our study, differential expression of *NOTCH2, Rac1* and *SMAD4* were all observed to be up-regulated after 6 h of LPS stimulation of *A. fangsiao* white body. This suggests that *NOTCH2, Rac1* and *SMAD4* may synergistically regulate immune cell migration, cell proliferation and inflammatory responses to attenuate the infectious effects of LPS. The focus of future research should be on further exploring the immunological functions these components serve in the white tissue of *A. fangsiao*.

## 5 Conclusion

Transcriptome-based protein-protein interaction analysis reveals the complexity and diversity of the *A. fangsiao* immune gene network. Our study confirms the significant effect of LPS stimulation on the immune mechanism of white body of *A. fangsiao*, further revealing the key pathways and genes involved in the infection and immune regulation process. In particular, we identified several important immune-related pathways, such as chemokine signaling pathway, Hippo signaling pathway, and Leukocyte transendothelial migration, as well as significant expression changes of hub genes, such as *AKT3, MAPK14*, and *PIK3CA*. These findings deepen our understanding of the immune mechanisms of *A. fangsiao* and provide new ideas and targets for future research and immunotherapy.

## Funding

This research was funded by the Ministry of Agriculture of the People's Republic of China with grant number CARS-49.

## CRediT authorship contribution statement

**Zhengcai Lu:** Writing – original draft. **Yancheng Zhao:** Methodology. **Tingjin Lv:** Methodology. **Xipan Chen:** Formal analysis. **Cuiju Cui:** Methodology. **Xiumei Liu:** Visualization. **Zan Li:** Writing – review & editing, Conceptualization. **Liyong Wang:** Writing – review & editing. **Xiaohui Xu:** Writing – review & editing, Conceptualization. **Jianmin Yang:** Project administration, Funding acquisition.

## Declaration of competing interest

The authors declare that they have no known competing financial interests or personal relationships that could have appeared to influence the work reported in this paper.

## Data Availability

Data will be made available on request.

## Appendix Supplementary materials

Image, application 1

Image, application 2

Image, application 3

Image, application 4

Image, application 5
