# Supplementary figures and images for: Transcriptome-based protein-protein interaction analysis reveals immune gene network elucidating white body immunity mechanisms in response to LPS stimulation in Amphioctopus fangsiao
Source: Comp Immunol Rep. 2024 Jun 12;7:200151. doi: 10.1016/j.cirep.2024.200151 (PMC11228953; doi:10.1016/j.cirep.2024.200151)

**Figure S1** Bioinformatics analysis process.


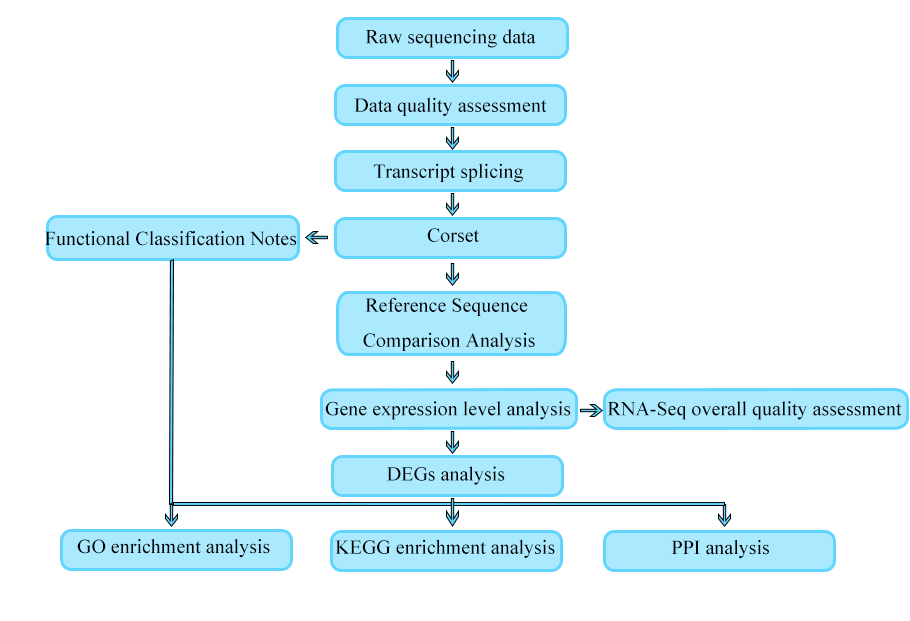

Supplement: Supplementary file 1 [file mmc1.docx]
